# Evaluation of genetic susceptibility between systemic lupus erythematosus and *GRB2* gene

**DOI:** 10.1038/s41598-019-46827-z

**Published:** 2019-07-17

**Authors:** Meifeng Xu, Yan Liu, Xiaoli Li, Chuantao Cheng, Yale Liu, Wei Dong, Shaoyi Du, Shengxiang Xiao

**Affiliations:** 1 Department of Dermatology, the Second Affiliated Hospital of Xi’an Jiao Tong University, Xi’an, Shaanxi China; 2 Institute of Artificial Intelligence and Robotics, Xi’an Jiaotong University, Xi’an, Shaanxi China

**Keywords:** Genetic predisposition to disease, Risk factors

## Abstract

Multiple lines of evidence have shown that systemic lupus erythematosus (SLE) is attributable to both genetic and environmental factors. The product of *GRB2* is a key factor in the activation of B cells and has been reported to be significantly associated with SLE in European populations. In the study, we aimed to investigate the relationship between *GRB2* and SLE. A total of 1,710 Han Chinese women comprising 567 SLE patients and 1,143 controls were recruited to genotype 20 selected tagging SNPs. We tested the potential association between 13 clinical variables of SLE and the significant polymorphisms related to SLE. The eQTL data were extracted from the GTEx database to examine the functional consequences of the targeted SNPs. A significant association signal was identified between rs36023980 and SLE in both genotypic and allelic analyses (OR = 0.61, *P* = 0.0003). Complement inhibition was shown to be significantly associated with the genotypes of SNP rs36023980 in SLE patients (*P*_genotype_ = 0.003). Further stratification analyses showed that the genetic association signal of SNP rs36023980 on SLE could only be identified in cases with complement inhibition. SNP rs36023980 was also identified to be significantly associated with the expression of *GRB2* in whole blood and sun-exposed skin. In conclusion, our findings confirm the results from the previous GWAS and are the first to report the association of *GRB2* with SLE in Han Chinese population.

## Introduction

Systemic lupus erythematosus (SLE) is an autoimmune disease characterized by inflammation of connective tissues^1^. SLE could lower the life expectancy of patients by significantly increasing the risk of multiple clinical manifestations including cardiovascular disease^2,3^, nephritis^4^, anemia^5^ etc. There is no cure for SLE, and common treatments such as NASIDs and corticosteroids may only relieve SLE symptoms^6^. The incidence rate of SLE is approximately 20–70 per 100,000 people globally and is related to ethnicity, age and gender^6^. In general, women are 9 times more often than men to be affected by SLE^6^. Europeans are at lower risk compared to other ethnicities, including Africans and Asians^6^.

Multiple lines of evidence have shown that SLE is a disease attributable to both genetic and environmental factors^4,7–11^. Early twin studies have shown that higher concordance was observed in monozygous twins than in dizygous twins^12^, and the heritability was estimated to be approximately 0.66^13^. Further gene association mapping studies have identified multiple susceptible loci of SLE. Since 2007, a total of 33 genome-wide association studies have reported more than 700 association signals from hundreds of susceptible loci of SLE^14^. A recent GWAS published in 2018 by Julià *et al*. has reported SNP rs36023980 of *GRB2* to be significantly associated with SLE disease status in European and Spanish populations^15^. In addition, Another bioinformatics study using genome molecular interaction networks have identified *GRB2* as a potential candidate gene for multiple autoimmune disorders including SLE^16^. *GRB2* encodes growth factor receptor-bound protein 2, which can form a complex with activated epidermal growth factor receptor (EGFR) and with the RAS-specific guanine nucleotide exchange factor SOS1, and thereby mediates the activation of RAS^17^. *GRB2* is a key factor for the activation of B cell related signaling pathways^18^. Despite the recent significant findings reported by GWAS, further replication studies are still needed to determine the association between *GRB2* and SLE in other populations to validate this previous GWAS hit. In addition, it is still not very clear of the functional significance for the SNP identified to be significantly associated with SLE disease status. It is more clinically valuable to examine the potential connections between polymorphisms of *GRB2* and clinical variables of SLE.

In the present study, we aimed to investigate the genetic association between polymorphisms of *GRB2* and SLE. A total of 1,710 Chinese women were recruited and 20 selected tagging SNPs were genotyped to examine the differences in distribution of genotypes in SLE patients and controls. We tested the potential association between 13 clinical variables of SLE and the significant polymorphism related to the disease status of SLE. eQTL analyses were also performed to explore the potential functional consequence of significant SNPs.

## Methods

### Study subjects

A total of 1,710 unrelated Han Chinese women comprising 567 SLE patients and 1,143 controls were recruited from the Second Affiliated Hospital of Xi’an Jiaotong University. All patients with SLE were diagnosed according to the 1997 American College of Rheumatology (ACR) classification criteria for SLE. Patients with other autoimmune diseases, cancer, systemic diseases, and other serious diseases were excluded from the study. Meanwhile, unrelated healthy controls were recruited from the same hospital. The inclusion criteria were as follows: (1) without autoimmune diseases, cancer, systemic diseases, and serious diseases and (2) without autoimmune diseases in members of their immediate family. All subjects were born in the local area. Subjects were randomly chosen, unrelated Han Chinese individuals without migration history, which ensures the genetic homogeneity in the study. Data on general characteristics and clinical information including malar rash, photosensitivity, leucopenia, anemia, complement depressed, renal disorder, neurologic disorder, arthritis, anti-dsDNA, anti-RNP, anti-Sm, anti-SSA and anti-SSB were obtained from medical records or questionnaire (Supplemental Table S1). No significant differences in age could be identified between cases and controls (*P* = 0.3903). Written informed consent was obtained from subjects.

This research was performed in accordance with the ethical guidelines of the Declaration of Helsinki (version 2002) and was approved by the Ethics Committee of Xi’an Jiaotong University.

### SNP selection and genotyping

SNPs located within the *GRB2* gene region, with a minor allele frequency (MAF) >0.05, were searched in the 1000-genomes CHB database. An r^2^ ≥0.8 was used as the cutoff criteria in pairwise tagging. Overall, 20 tagging SNPs were selected for further genotyping. The basic information for these 20 tagging SNPs is summarized in Supplemental Table S1. All of these 20 SNPs were intronic SNPs of *GRB2*. Genomic DNA was extracted from peripheral blood leukocytes according to the manufacturer’s protocol (Genomic DNA kit, Axygen Scientific Inc., CA, USA). The selected tagging SNPs were genotyped using the high-throughput Sequenom MassARRAY platform (Sequenom, San Diego, CA, USA) according to the manufacturer’s protocol. The results were processed using Sequenom Typer 4.0 software to generate genotypic data. For quality control, the disease state of the sample was unknown throughout the genotyping process. The final genotyping call rate for each SNP was greater than 99%, and the overall genotyping call rate was 99.9%. Subsequently, we randomly selected 5% of the samples for regenotyping, and the results were exactly the same as before.

### Statistical analyses

Hardy-Weinberg equilibrium (HWE) tests were performed for each SNP in the control group (Supplemental Table S2). Logistic models were fitted for each SNP and age was included as a main covariate to adjust using Plink^19^. Linkage disequilibrium (LD) blocks were constructed by Haploview based on the algorithm proposed by Gabriel *et al*.^20^. Haplotype-based analyses were then performed for each LD block. Bonferroni corrections were applied to address multiple comparisons. For single-marker-based association analyses, the threshold used for significance of *P* values was 0.05/20 = 0.0025. In addition to genetic association analyses, we also examined the distributions of multiple clinical variables among different genotypic groups of SLE patients. Power analyses were performed by using GAS power calculator (http://csg.sph.umich.edu/abecasis/gas_power_calculator/index.html). For the present study, we could achieve 80% statistical power for a SNP with effect size equal or greater than 1.5 (Supplemental Figure S1).

### Bioinformatics and eQTL analyses

To further investigate the functional consequences of SNPs significantly associated with SLE disease status, we extracted eQTL data of 47 human tissues from the GTEx database (https://gtexportal.org/home/)^21^. Differences in *GRB2* gene expression levels among different genotypic groups of targeted SNPs were examined and significant eQTL signals in specific human tissues were reported.

## Results

### Association between SNP rs36023980 and SLE disease status

**Figure 1:**
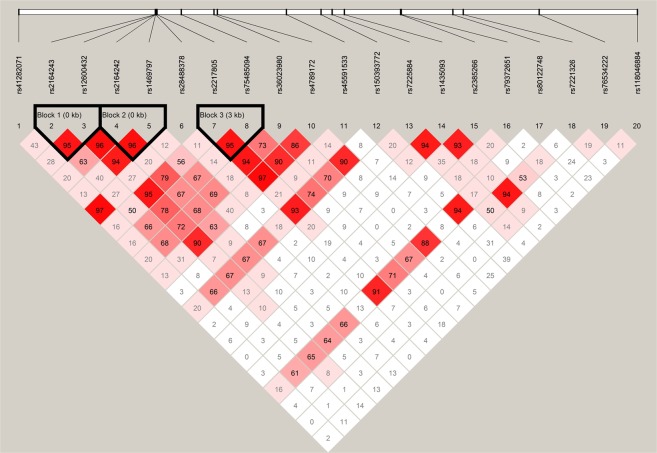
The LD structure of the selected SNPs. D’ values is indicated in each square.

A significant association signal was identified between SNP rs36023980 and SLE disease status in both genotypic and allelic analyses (Table 1, Supplemental Table S3). The minor allele of rs36023980, T allele, could have a protective effect on SLE (OR = 0.61, *P* = 0.0003). Three LD blocks were constructed (Fig. 1). None of these LD blocks were significantly associated with SLE in the following haplotype-based analyses (Supplemental Table S4).

**Table 1:** Differences in distribution of genotypes and alleles for rs36023980 between SLE patients and controls.

	Genotypic Tests				Allelic Tests				
	TT (N = 19)	CT (N = 267)	CC (N = 1,424)	*P	T (N = 305)	C (N = 3,115)	**T	**OR	**P
SLE Patients (fre%)	3 (0.5%)	66 (11.6%)	498 (87.9%)	0.0011	72 (6.3%)	1,062 (93.7%)	−3.62	0.61	0.0003
Controls (fre%)	16 (1.4%)	201 (17.6%)	926 (81%)		233 (10.2%)	2,053 (89.8%)			

Fre: frequency.

*Fisher exact test was performed due to the sparse cells.

**T statistics, OR and *P* values obtained from logistic model adjusted for age were reported.

### Association between SNP rs36023980 and SLE related clinical features

Complement inhibition was shown to be significantly associated with the genotypes of SNP rs36023980 in SLE patients (*P*_*adjusted*_ = 0.003, Table 2). None of the other clinical variables were determined to be significantly associated with this SNP. The C allele of rs36023980 may significantly lower the rate for complement inhibition by approximately 50% (OR = 0.48). To further examine whether the association of the rs36023980 with SLE risk is independent of the complement status, we performed stratified logistic regression analyses. The results were summarized in Table 3. As we can see, the association signal of rs36023980 was mainly derived from SLE cases with complement depression (OR = 0.47,*P* = 1.44 × 10^−5^).

**Table 2:** Association between SNP rs36023980 and clinical variables in SLE patients.

	Genotypic Tests				Allelic Tests				
	CC	CT	TT	*P	C	T	Chi	**P	**OR
Malar rash									
*Yes*	349	39	1		737	41			
*No*	149	27	2	0.0745	325	31	4.8550	0.0325	1.69
Photosensitivity									
*Yes*	214	27	2		455	31			
*No*	284	39	1	0.6939	607	41	0.0012	0.9925	1.00
Leucopenia									
*Yes*	197	24	1		418	26			
*No*	301	42	2	0.8640	644	46	0.2987	0.5794	1.15
Anemia									
*Yes*	223	31	0		477	31			
*No*	275	35	3	0.3615	585	41	0.0943	0.7562	1.08
**Complement inhibition**									
***Yes***	**123**	**24**	**3**		**270**	**30**			
***No***	**375**	**42**	**0**	**0.0030**	**792**	**42**	**9.1430**	**0.0030**	**0.48**
Renal disorder									
*Yes*	243	36	2		522	40			
*No*	255	30	1	0.5462	540	32	1.1060	0.3169	0.78
Neurologic disorder									
*Yes*	377	49	3		803	55			
*No*	121	17	0	0.8466	259	17	0.0221	0.8610	0.95
Arthritis									
*Yes*	198	25	1		421	27			
*No*	300	41	2	0.9090	641	45	0.1295	0.7212	1.09
Anti-dsDNA									
*Yes*	251	33	1		535	35			
*No*	247	33	2	0.9607	527	37	0.0841	0.7846	1.07
Anti-RNP									
*Yes*	279	37	3		595	43			
*No*	219	29	0	0.4333	467	29	0.3743	0.5522	0.86
Anti-Sm									
*Yes*	295	41	2		631	45			
*No*	203	25	1	0.8665	431	27	0.2663	0.6143	0.88
Anti-SSA									
*Yes*	179	24	1		382	26			
*No*	319	42	2	1.0000	680	46	0.0006	0.9898	1.00
Anti-SSB									
*Yes*	367	54	2		788	58			
*No*	131	12	1	0.3085	274	14	1.4380	0.2337	0.70

*Fisher exact tests were performed because of sparse cells. Significant findings are highlighted in bold. The threshold of *P* values is 0.05/13≈0.004.

**OR and P values were obtained from logistic model with age as a covariate.

**Table 3:** Genetic association analyses for rs36023980 stratified by complement inhibition.

CHR	SNP	BP	A1	Complement inhibition (N cases = 417)			No complement inhibition (N cases = 150)		
				OR	*T*	*P*	OR	*T*	*P*
17	rs36023980	73341284	T	0.47	−4.34	1.44 × 10^−5^	0.98	−0.09	0.9309

### eQTL pattern of SNP rs36023980

Using the data set extracted from the GTEx database, we demonstrate that *SNP rs36023980* is significantly associated with the gene expression levels of *GRB2* in two types of human tissues: whole blood (*P* = 7.7 × 10^−6^) and sun-exposed skin (*P* = 0.0003) (Supplemental Table S5, Supplemental Figure S2). The C allele of rs36023980 can significantly increase the gene expression levels of *GRB2* in both tissues.

## Discussion

With the fast development and application of sequencing and genetic association analyses for studying genetic susceptibility of complex diseases, candidate gene-based association studies have successfully identified susceptibility loci for many complex diseases^22–36^. In this study, we have identified a significant signal association between *GRB2* and disease status of SLE. Our findings are a successful replication of a recent GWAS meta-analysis conducted by Julia *et al*., in which SNP rs36023980 of *GRB2* was found to be significantly associated with disease status of SLE in a combined dataset of European and Spanish populations. Our study and this GWAS agree to the direction of the effect of SNP rs36023980. Both studies identified the C allele of this SNP as a risk allele and the T allele as a protective allele. To the best of our knowledge, our study is the first to identify the genetic association between *GRB2* and SLE in Chinese Han populations.

*GRB2* encodes a receptor tyrosine-kinase (RTK) adaptor protein comprising one SH2 and two SH3 domains^37^. The RTK adaptor can bind to EGFR and can be involved with the activation of RAS^16^. An animal study using mice has shown that *Grb2* could regulate B-cell maturation and memory responses^17^. The Grb2-dependent signaling pathways are crucial for control of secondary humoral immune responses^17^. Additional studies have shown that Grb2 is also very important for T Cell development and differentiation^38^.

SNP rs36023980 is located at the intronic region of *GRB2* and therefore could not have significant functional consequences by altering the structure of the protein encoded by *GRB2*. Nevertheless, this SNP might play an important role in regulating the gene expression of *GRB2*. Using data extracted from the GTEx database, our eQTL analyses show that this SNP is significantly associated with the gene expression level of *GRB2* in two types of human tissues: whole blood and exposed skin of the lower legs. This finding provides evidence for the functional consequences of SNP rs36023980 to *GRB2*. SNP rs36023980 located in a region enriched in CG and might involve a CpG site (change T/C). Therefore this SNP might affect the DNA methylation pattern in the region. However, these findings are insufficient to determine whether this signal is a direct or indirect association In this study, we genotyped only 20 intronic SNPs of *GRB2*. The genetic marker coverage in previous GWAS was even lower than the present study. Thus, rs36023980 may be just a surrogate of some underlying ungenotyped variants that are in strong LD with rs36023980. For future research, a sequencing-based study design would increase the coverage of genetic information and offer a more thorough genetic scan for associations between *GRB2* and SLE.

In addition to the association analyses conducted for disease status, we examined the association between multiple clinical variables related to SLE and SNP rs36023980 in our patients. Among all of the 13 clinical variables, only complement inhibition was significantly associated with the genotype of rs36023980 after being corrected for multiple comparisons. The complement system, which is part of the innate immune system, enhances the ability of antibodies and promotes inflammation. Previous studies have indicated that complement system plays a major role in SLE^39^. Genetic studies have identified that the genetic deficiencies of many classical complement related pathway components are significantly associated with SLE status^40^. SLE is an autoimmune disorder characterized by widespread complement activation and deposition of complement fragments in the kidney^1^. In this sense, the direction of effect of the C allele of rs36023980 identified here was in accordance with its effect for SLE disease risk. The C allele was a risk allele of SLE disease status. Specifically, in SLE patients, the C allele of rs36023980 was related to no complement inhibition and therefore was related to increased complement activation. It seems that the genetic effect of SNP rs36023980 on SLE was mediated through complement inhibition based on the results from our stratification analyses. However, we need to be careful to interpret this result because our limited sample size (only 150 cases in the group of no complement inhibition). More research are needed in the future.

Our study suffers from several limitations. First, only women were enrolled in this study. Despite the sex ratio of 9:1 (female to male) for SLE, excluding male patients would harm the generalization of our study results. Second, population stratification might be a potential confounder for our significant hit. This might be even worse considering the fact that the Chinese Han population is highly genetically diverse. In addition, only SNPs located within the gene region of *GRB2* were included in this study because of our limited funds. Multiple lines of evidence have shown that important regulatory regions are within 20 kb up- and downstream the gene region. In future studies, SNPs located within these important regulatory regions should be included.

In sum, we have identified a significant association between SNP rs36023980 of *GBR2* and SLE disease status. This SNP was also identified to be significantly associated with clinical features of SLE patients. Our findings confirm the results from the previous GWAS and are the first to report the association of *GRB2* with SLE in Han Chinese population.

## Supplementary information

Supplemental Materials
