# Supplementary material for: Evaluation of genetic susceptibility between systemic lupus erythematosus and GRB2 gene
Source: Sci Rep. 2019 Jul 17;9:10335. doi: 10.1038/s41598-019-46827-z (PMC6637148; doi:10.1038/s41598-019-46827-z)
Supplement: Supplementary file 1 — Supplemental Materials [file 41598_2019_46827_MOESM1_ESM.docx]

***Title***: Evaluation of genetic susceptibility between systemic lupus erythematosus and *GRB2* gene

***Author names and affiliations***: Meifeng Xu^1^, Yan Liu^1^, Xiaoli Li^1^, Chuantao Cheng^1^, Yale Liu^1^, Wei Dong^1^, Shaoyi Du^2^ and Shengxiang Xiao^1^

^1^ Department of Dermatology, the Second Affiliated Hospital of Xi'an Jiao Tong University, Xi'an, Shaanxi, China;

^2^ Institute of Artificial Intelligence and Robotics, Xi’an Jiaotong University, Xi’an Shaanxi, China

***Corresponding Author***:

Shaoyi Du, Institute of Artificial Intelligence and Robotics, Xi’an Jiaotong University, 28 Xianning West Road, Beilin District, Xi’an, 710049,China.

Tel: 86-29-82668672; Fax: 86-29-82668672; E-mail: shaoyiduiai@163.com

Shengxiang Xiao, Department of Dermatology, the Second Affiliated Hospital of Xi’an Jiaotong University, 157 Xiwu Road, Xincheng District, Xi'an, 710004, China.

Tel: 86-29-87679329; Fax: 86-29-87679329; E-mail: shxaxiaoxjtu@163.com

Supplemental Table S1. The clinical characteristics of the study subjects.

| Characteristics | Subjects (N = 1,710) | | *P*-value |
| --- | --- | --- | --- |
|  | Patients (N=567) | Controls (N=1,143) |  |
| Age (years), mean±SD | 38.3±8.6 | 38.7±8.7 | 0.3903 |
| Malar rash (Yes/No,%) | 178/389 (31/69) | - | - |
| Photosensitivity (Yes/No,%) | 324/243 (57/43) | - | - |
| Leucopenia (Yes/No,%) | 345/222 (61/39) | - | - |
| Anemia (Yes/No,%) | 313/254 (55/45) | - | - |
| Complement depression (Yes/No,%) | 417/150 (74/26) | - | - |
| Renal disorder (Yes/No,%) | 286/281 (50/50) | - | - |
| Neurologic disorder (Yes/No,%) | 138/429 (24/76) | - | - |
| Arthritis (Yes/No,%) | 343/224 (60/40) | - | - |
| Anti-dsDNA (Yes/No,%) | 282/285 (50/50) | - | - |
| Anti-RNP (Yes/No,%) | 248/319 (44/56) | - | - |
| Anti-Sm (Yes/No,%) | 229/338 (40/60) | - | - |
| Anti-SSA (Yes/No,%) | 363/204 (64/36) | - | - |
| Anti-SSB (Yes/No,%) | 144/423 (25/75) | - | - |

SD: standard deviation.

Supplemental Table S2. MAF and HWE results for the candidate SNPs selected for genotyping.

| CHR | POS | SNP | Alleles | Function | Gene | MAF | HWE |
| --- | --- | --- | --- | --- | --- | --- | --- |
| 17 | 75320799 | rs41282071 | C/G | intron | *GRB2* | 0.18 | 0.32 |
| 17 | 75337870 | rs2164243 | A/T | intron | *GRB2* | 0.26 | 0.94 |
| 17 | 75337901 | rs12600432 | C/T | intron | *GRB2* | 0.08 | 1.00 |
| 17 | 75337904 | rs2164242 | A/G | intron | *GRB2* | 0.12 | 0.32 |
| 17 | 75337907 | rs1469797 | A/G | intron | *GRB2* | 0.16 | 0.28 |
| 17 | 75337916 | rs28488378 | C/T | intron | *GRB2* | 0.26 | 1.00 |
| 17 | 75338083 | rs2217805 | A/G | intron | *GRB2* | 0.44 | 0.47 |
| 17 | 75341118 | rs75485094 | A/G | intron | *GRB2* | 0.10 | 0.29 |
| 17 | 75345203 | rs36023980 | C/T | intron | *GRB2* | 0.09 | 0.19 |
| 17 | 75345631 | rs4789172 | C/T | intron | *GRB2* | 0.27 | 0.94 |
| 17 | 75354264 | rs45591533 | G/T | intron | *GRB2* | 0.09 | 0.85 |
| 17 | 75358592 | rs150393772 | C/T | intron | *GRB2* | 0.07 | 0.53 |
| 17 | 75359979 | rs7225884 | A/T | intron | *GRB2* | 0.17 | 0.39 |
| 17 | 75361505 | rs1435093 | A/G | intron | *GRB2* | 0.06 | 0.77 |
| 17 | 75368544 | rs2385266 | A/C | intron | *GRB2* | 0.21 | 0.93 |
| 17 | 75368628 | rs79372651 | C/T | intron | *GRB2* | 0.06 | 1.00 |
| 17 | 75375077 | rs80122748 | G/T | intron | *GRB2* | 0.06 | 0.26 |
| 17 | 75376375 | rs7221326 | A/C | intron | *GRB2* | 0.11 | 0.76 |
| 17 | 75385872 | rs76534222 | C/T | intron | *GRB2* | 0.06 | 0.39 |
| 17 | 75398162 | rs118046884 | C/T | intron | *GRB2* | 0.13 | 0.45 |

CHR: chromosome; POS: position; MAF: minor allele frequency; HWE: *P* values for Hardy-Weinberg Equilibrium tests.

Supplemental Table S3. Full results for association analyses of the 20 selected SNPs using logistic models.

| CHR | SNP | POS |  | Additive Model | | | Dominant Model | | | Recessive Model | | |
| --- | --- | --- | --- | --- | --- | --- | --- | --- | --- | --- | --- | --- |
|  |  |  | A1 | OR | *T* | *P* | OR | *T* | *P* | OR | *T* | *P* |
| 17 | rs41282071 | 73316880 | G | 1.03 | 0.33 | 0.7389 | 1.04 | 0.34 | 0.7333 | 1.04 | 0.13 | 0.8962 |
| 17 | rs2164243 | 73333951 | A | 1.02 | 0.19 | 0.8521 | 1.02 | 0.17 | 0.8640 | 1.03 | 0.12 | 0.9037 |
| 17 | rs12600432 | 73333982 | T | 0.90 | -0.79 | 0.4282 | 0.90 | -0.75 | 0.4522 | 0.76 | -0.41 | 0.6817 |
| 17 | rs2164242 | 73333985 | T | 0.95 | -0.45 | 0.6554 | 0.96 | -0.31 | 0.7538 | 0.67 | -0.70 | 0.4860 |
| 17 | rs1469797 | 73333988 | T | 1.05 | 0.48 | 0.6342 | 1.04 | 0.35 | 0.7300 | 1.21 | 0.58 | 0.5650 |
| 17 | rs28488378 | 73333997 | C | 0.97 | -0.34 | 0.7332 | 0.98 | -0.19 | 0.8481 | 0.91 | -0.47 | 0.6403 |
| 17 | rs2217805 | 73334164 | C | 0.97 | -0.48 | 0.6337 | 0.95 | -0.50 | 0.6136 | 0.97 | -0.26 | 0.7948 |
| 17 | rs75485094 | 73337199 | A | 1.10 | 0.80 | 0.4227 | 1.11 | 0.76 | 0.4498 | 1.23 | 0.45 | 0.6522 |
| 17 | rs36023980 | 73341284 | T | 0.61 | -3.62 | 0.0003 | 0.59 | -3.54 | 0.0004 | 0.38 | -1.55 | 0.1220 |
| 17 | rs4789172 | 73341712 | T | 1.03 | 0.41 | 0.6784 | 1.02 | 0.22 | 0.8271 | 1.12 | 0.58 | 0.5627 |
| 17 | rs45591533 | 73350345 | G | 0.96 | -0.31 | 0.7586 | 0.96 | -0.29 | 0.7725 | 0.90 | -0.17 | 0.8642 |
| 17 | rs150393772 | 73354673 | T | 0.94 | -0.48 | 0.6284 | 0.94 | -0.43 | 0.6694 | 0.76 | -0.39 | 0.6931 |
| 17 | rs7225884 | 73356060 | A | 1.05 | 0.47 | 0.6383 | 1.04 | 0.34 | 0.7343 | 1.20 | 0.57 | 0.5715 |
| 17 | rs1435093 | 73357586 | G | 1.25 | 1.51 | 0.1324 | 1.24 | 1.38 | 0.1671 | 2.03 | 1.00 | 0.3183 |
| 17 | rs2385266 | 73364625 | T | 0.98 | -0.20 | 0.8416 | 0.95 | -0.47 | 0.6396 | 1.14 | 0.53 | 0.5970 |
| 17 | rs79372651 | 73364709 | T | 0.93 | -0.45 | 0.6519 | 0.92 | -0.48 | 0.6325 | 1.03 | 0.03 | 0.9766 |
| 17 | rs80122748 | 73371158 | T | 1.11 | 0.71 | 0.4765 | 1.11 | 0.65 | 0.5128 | 1.35 | 0.47 | 0.6402 |
| 17 | rs7221326 | 73372456 | A | 0.96 | -0.37 | 0.7105 | 0.97 | -0.23 | 0.8147 | 0.71 | -0.64 | 0.5206 |
| 17 | rs76534222 | 73381953 | C | 1.24 | 1.49 | 0.1371 | 1.23 | 1.33 | 0.1821 | 2.01 | 1.10 | 0.2719 |
| 17 | rs118046884 | 73394243 | C | 0.92 | -0.75 | 0.4548 | 0.92 | -0.69 | 0.4925 | 0.83 | -0.48 | 0.6332 |

CHR: chromosome; POS: position; A1: minor allele.

Supplemental Table S4. Summarized results for haplotype-based analyses.

| LOCUS | HAPLOTYPE | F_A | F_U | χ^2^ | DF | *P* | SNPS |
| --- | --- | --- | --- | --- | --- | --- | --- |
| *GRB2* | OMNIBUS | - | - | 1.39 | 2.00 | 0.50 | rs2164243\|rs12600432 |
| *GRB2* | AT | 0.08 | 0.09 | 0.80 | 1.00 | 0.37 | rs2164243\|rs12600432 |
| *GRB2* | AC | 0.18 | 0.17 | 0.78 | 1.00 | 0.38 | rs2164243\|rs12600432 |
| *GRB2* | TC | 0.74 | 0.74 | 0.04 | 1.00 | 0.84 | rs2164243\|rs12600432 |
| *GRB2* | OMNIBUS | - | - | 3.82 | 2.00 | 0.15 | rs2164242\|rs1469797 |
| *GRB2* | TT | 0.11 | 0.12 | 0.48 | 1.00 | 0.49 | rs2164242\|rs1469797 |
| *GRB2* | CT | 0.06 | 0.05 | 3.53 | 1.00 | 0.06 | rs2164242\|rs1469797 |
| *GRB2* | CC | 0.83 | 0.84 | 0.28 | 1.00 | 0.59 | rs2164242\|rs1469797 |
| *GRB2* | OMNIBUS | - | - | 1.02 | 2.00 | 0.60 | rs2217805\|rs75485094 |
| *GRB2* | CA | 0.10 | 0.09 | 0.50 | 1.00 | 0.48 | rs2217805\|rs75485094 |
| *GRB2* | CG | 0.34 | 0.36 | 0.76 | 1.00 | 0.38 | rs2217805\|rs75485094 |
| *GRB2* | TG | 0.56 | 0.55 | 0.18 | 1.00 | 0.67 | rs2217805\|rs75485094 |

F_A: frequency of haplotype in cases; F_U: frequency of haplotype in controls; DF: degrees of freedom.

Supplemental Table S5. Results of eQTL analyses for rs36023980 on *GRB2* in multiple human tissues.

| Gene | SNP | *P* | A1 | NES | *T*-statistic | Tissue |
| --- | --- | --- | --- | --- | --- | --- |
| ***GRB2*** | **rs36023980** | **7.7×10^-6^** | **C** | **0.085** | **4.60** | **Whole Blood** |
| ***GRB2*** | **rs36023980** | **0.0003** | **C** | **0.110** | **3.70** | **Skin - Exposed to Sun (Lower leg)** |
| *GRB2* | rs36023980 | 0.0017 | C | 0.100 | 3.20 | Adipose - Subcutaneous |
| *GRB2* | rs36023980 | 0.0018 | C | 0.075 | 3.10 | Thyroid |
| *GRB2* | rs36023980 | 0.0025 | C | 0.100 | 3.10 | Skin - Not Exposed to Sun (Suprapubic) |
| *GRB2* | rs36023980 | 0.0039 | C | 0.170 | 3.00 | Brain - Cerebellar Hemisphere |
| *GRB2* | rs36023980 | 0.0044 | C | 0.110 | 2.90 | Breast - Mammary Tissue |
| *GRB2* | rs36023980 | 0.0046 | C | 0.087 | 2.90 | Nerve - Tibial |
| *GRB2* | rs36023980 | 0.007 | C | -0.140 | -2.80 | Uterus |
| *GRB2* | rs36023980 | 0.014 | C | -0.075 | -2.50 | Esophagus - Mucosa |
| *GRB2* | rs36023980 | 0.022 | C | 0.060 | 2.30 | Lung |
| *GRB2* | rs36023980 | 0.022 | C | 0.056 | 2.30 | Testis |
| *GRB2* | rs36023980 | 0.03 | C | 0.099 | 2.20 | Brain - Hypothalamus |
| *GRB2* | rs36023980 | 0.034 | C | -0.140 | -2.20 | Pituitary |
| *GRB2* | rs36023980 | 0.036 | C | -0.130 | -2.10 | Brain - Putamen (basal ganglia) |
| *GRB2* | rs36023980 | 0.045 | C | 0.082 | 2.00 | Colon - Transverse |
| *GRB2* | rs36023980 | 0.046 | C | 0.094 | 2.00 | Artery - Aorta |
| *GRB2* | rs36023980 | 0.059 | C | 0.140 | 1.90 | Brain - Amygdala |
| *GRB2* | rs36023980 | 0.07 | C | 0.050 | 1.80 | Adipose - Visceral (Omentum) |
| *GRB2* | rs36023980 | 0.079 | C | -0.089 | -1.80 | Brain - Hippocampus |
| *GRB2* | rs36023980 | 0.085 | C | 0.070 | 1.70 | Heart - Atrial Appendage |
| *GRB2* | rs36023980 | 0.13 | C | 0.073 | 1.50 | Brain - Cerebellum |
| *GRB2* | rs36023980 | 0.14 | C | 0.083 | 1.50 | Artery - Coronary |
| *GRB2* | rs36023980 | 0.26 | C | 0.041 | 1.10 | Stomach |
| *GRB2* | rs36023980 | 0.26 | C | 0.078 | 1.10 | Vagina |
| *GRB2* | rs36023980 | 0.28 | C | 0.050 | 1.10 | Brain - Cortex |
| *GRB2* | rs36023980 | 0.3 | C | 0.048 | 1.00 | Brain - Caudate (basal ganglia) |
| *GRB2* | rs36023980 | 0.33 | C | -0.040 | -0.99 | Liver |
| *GRB2* | rs36023980 | 0.38 | C | 0.033 | 0.87 | Colon - Sigmoid |
| *GRB2* | rs36023980 | 0.38 | C | -0.046 | -0.89 | Ovary |
| *GRB2* | rs36023980 | 0.48 | C | -0.024 | -0.71 | Artery - Tibial |
| *GRB2* | rs36023980 | 0.55 | C | -0.022 | -0.60 | Muscle - Skeletal |
| *GRB2* | rs36023980 | 0.57 | C | -0.013 | -0.57 | Cells - Transformed fibroblasts |
| *GRB2* | rs36023980 | 0.62 | C | 0.024 | 0.50 | Heart - Left Ventricle |
| *GRB2* | rs36023980 | 0.63 | C | -0.032 | -0.48 | Cells - EBV-transformed lymphocytes |
| *GRB2* | rs36023980 | 0.65 | C | -0.034 | -0.46 | Brain - Spinal cord (cervical c-1) |
| *GRB2* | rs36023980 | 0.68 | C | 0.011 | 0.42 | Esophagus - Muscularis |
| *GRB2* | rs36023980 | 0.71 | C | 0.020 | 0.38 | Spleen |
| *GRB2* | rs36023980 | 0.73 | C | -0.031 | -0.35 | Brain - Substantia nigra |
| *GRB2* | rs36023980 | 0.77 | C | -0.023 | -0.29 | Minor Salivary Gland |
| *GRB2* | rs36023980 | 0.77 | C | 0.015 | 0.29 | Pancreas |
| *GRB2* | rs36023980 | 0.78 | C | -0.015 | -0.28 | Adrenal Gland |
| *GRB2* | rs36023980 | 0.83 | C | -0.011 | -0.22 | Brain - Nucleus accumbens (basal ganglia) |
| *GRB2* | rs36023980 | 0.84 | C | -0.010 | -0.20 | Small Intestine - Terminal Ileum |
| *GRB2* | rs36023980 | 0.92 | C | 0.004 | 0.10 | Brain - Anterior cingulate cortex (BA24) |
| *GRB2* | rs36023980 | 0.95 | C | -0.003 | -0.06 | Brain - Frontal Cortex (BA9) |
| *GRB2* | rs36023980 | 1 | C | -0.0002 | -0.004 | Prostate |

NES: normalized effect size. The threshold of *P* values is 0.05/47≈0.001.


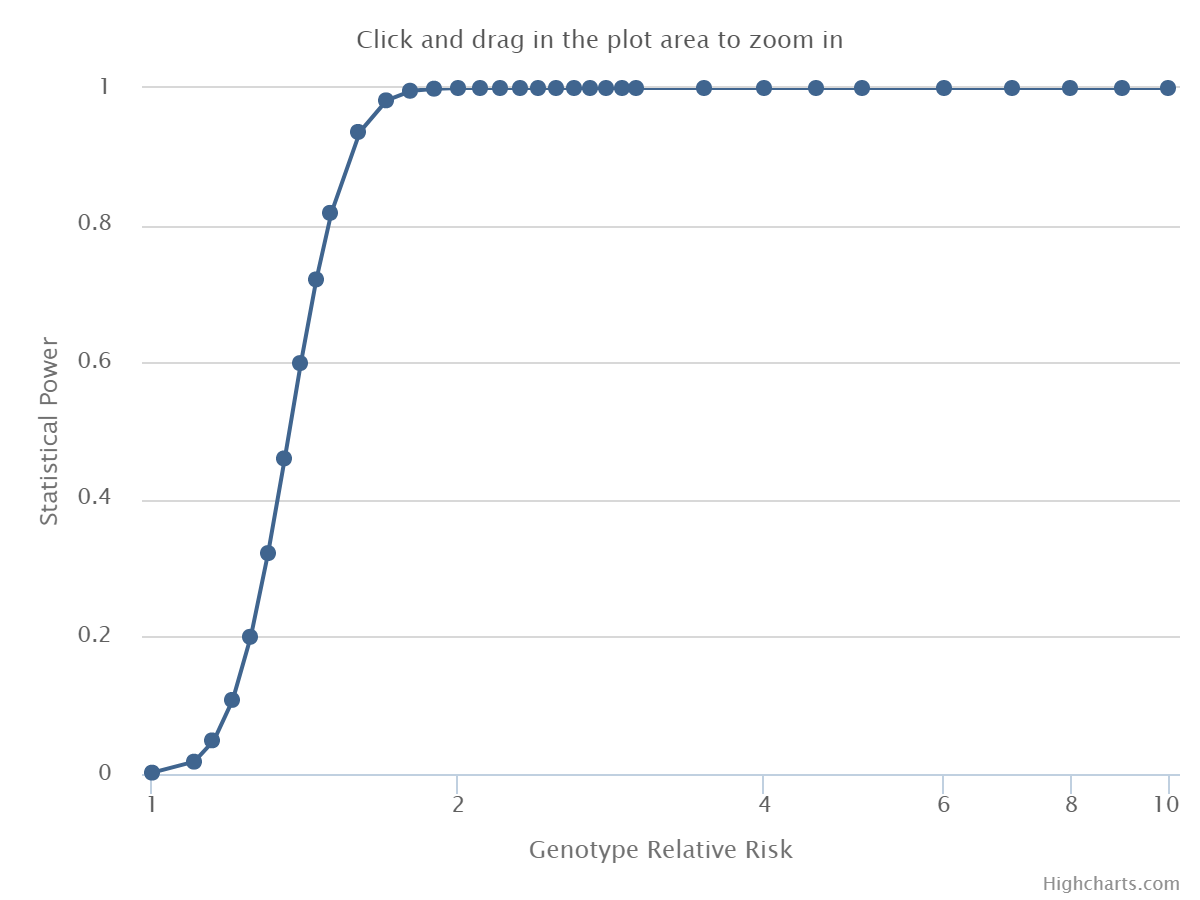


Supplemental Figure S1. Results of power analyses for the present study.


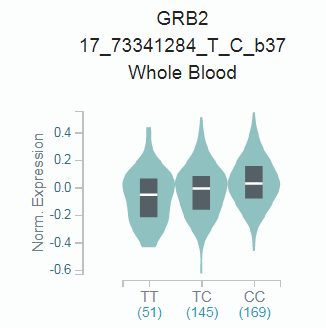


Supplemental Figure S2. Violin plot of gene expression of *GRB2* in whole blood classified by genotypes of SNP rs36023980.
